# Leukotriene B_4_, administered via intracerebroventricular injection, attenuates the antigen-induced asthmatic response in sensitized guinea pigs

**DOI:** 10.1186/1742-2094-7-12

**Published:** 2010-02-11

**Authors:** Yi-Liang Zhu, Shui-Jun Zhang, Yang-Mei Deng, Xin-Wei Dong, Jun-Xia Jiang, Qiang-Min Xie

**Affiliations:** 1 Zhejiang Respiratory Drugs Research Laboratory of State Food and Drug Administration of China, Medical Science College of Zhejiang University, Hangzhou, China; 2 Department of Pharmacology, Zhejiang Medical College, Hangzhou, China

## Abstract

**Background:**

Despite intensive studies focused on the pathophysiology of asthmatic inflammation, little is known about how cross-talk between neuroendocrine and immune systems regulates the inflammatory response during an asthmatic attack. We recently showed corresponding changes of cytokines and leukotriene B_4 _(LTB_4_) in brain and lung tissues of antigen-challenged asthmatic rats. Here, we investigated how LTB_4 _interacts with the neuroendocrine-immune system in regulating antigen-induced asthmatic responses in sensitized guinea pigs.

**Methods:**

Ovalbumin-sensitized guinea pigs were challenged by inhalation of antigen. Vehicle, LTB_4 _or U75302 (a selective LTB_4 _BLT1 receptor inhibitor) was given via intracerebroventricular injection (i.c.v.) 30 min before challenge. Airway contraction response was evaluated using Penh values before and after antigen challenge. The inflammatory response in lung tissue was evaluated 24 h after challenge. The LTB_4 _content of lung and brain homogenate preparations was detected by reversed phase high-performance liquid chromatography (RP-HPLC). Plasma levels of adrenocorticotropic hormone (ACTH) and corticosterone (CORT) were measured using ELISA kits.

**Results:**

Antigen challenge impaired pulmonary function and increased inflammatory cell infiltration in lung tissue. These responses could be significantly suppressed by LTB_4_, 30 ng i.c.v., in ovalbumin-sensitized guinea pigs. LTB_4 _content of lung and brain homogenates from antigen-challenged guinea pigs was significantly increased. In addition, administration of LTB_4 _via i.c.v. markedly increased CORT and ACTH level in plasma before antigen challenge, and there were further increases in CORT and ACTH levels in plasma after antigen challenge. U75302, 100 ng i.c.v., completely blocked the effects of LTB_4_. In addition, U75302, 100 ng via i.c.v. injection, markedly decreased LTB_4 _content in lung homogenates, but not in brain homogenates.

**Conclusions:**

Increased LTB_4 _levels in brain during asthmatic attacks down-regulates airway contraction response and inflammation through the BLT1 receptor. Stimulation of the hypothalamic-pituitary-adrenal axis by LTB_4 _may result in an increase in systemic glucocorticoids which, in turn, would feed back to suppress the asthmatic response.

## Background

Asthma is a result of pathological airway inflammation. Infiltrating inflammatory cells release mediators that contribute to manifestations of the disease. Importantly, these mediators cause activation of the stress system, which co-ordinates adaptive responses of the organism to stressors, maintaining basal and stress-related homeostasis. The stress system influences the activity of many other body systems, including the central nervous, cardiorespiratory, metabolic, endocrine, and immune systems, the functions of which are closely intertwined [1,2]. A major component of the stress system is the hypothalamic-pituitary-adrenal (HPA) axis. Stimulation of this axis by inflammatory mediators such as tumor necrosis factor-*α *(TNF-*α*), interleukin-1 (IL-1), IL-6, or histamine results in an increase in systemic glucocorticoids (corticosterone or cortisol in rodents and primates, respectively) which, in turn, feeds back to suppress immune and inflammatory reactions [3]. This suppressive activity includes the anti-inflammatory effects of glucocorticoids on airways. Leukotrienes (LTs) and other metabolites of 5-lipoxygenase (5-LO) pathways are known to play crucial roles in inflammatory pathways. However, the functional role of leukotrienes (LTs) and metabolites of 5-LO pathways on the neuro-endocrine-immune (NEI) network is still unclear.

Leukotriene (LT) B_4 _is a metabolic product of LTA_4 _resulting from the activity of LTA_4 _hydrolase (LTA_4_-H) in the 5-LO pathway. It is a potent leukocyte chemoattractant and activator that plays an important role in modulating immune responses [4]. LTB_4 _can be synthesized by various cell types both in the periphery and in the central nervous system (CNS) - for example mast cells, neutrophils [5], alveolar macrophages [6,7] and epithelial lens cells [8] in the periphery; and cultured primary rat astrocytes [9], neuronal cells [10], and glial cells [11] of the CNS - upon challenge with a variety of stimuli including LTB_4 _itself. LTB_4 _serves as a potent inflammatory mediator through the high affinity LTB_4 _receptor-1 (BLT1) on target cells. Various airway allergic and inflammatory diseases, including asthma [12-15], allergic rhinitis [16], idiopathic pulmonary fibrosis [17], acute lung injury or adult respiratory distress syndrome [18], and chronic obstructive pulmonary disease [19], are associated with increased levels of LTB_4 _and/or BLT1 expression. In some of these diseases, LTB_4 _levels can reflect disease activity and are reduced after treatment [4].

To date, it is unclear if metabolites of 5-LO pathways in the central nervous system regulate inflammatory responses in lung tissue during an asthma attack. Our previous studies have shown that the Th1/Th2 paradigm (ratio of interferon [IFN]-γ to interleukin [IL]-4) decreases [20], and the content of LTB_4 _increases, in CNS in concert with corresponding changes in bronchoalveolar lavage fluid (BALF) or lung tissue in ovalbumin-sensitized and challenged rats [21]. Also, the expressions of 5-LO and LTA_4_-H mRNA in cerebral cortex of asthmatic rats are significantly higher than those of control rats [22]. These findings suggest that LTs and proinflammatory cytokines in the central nervous system play a role in the pathogenesis of asthma in rats. In this study, we further explored how LTB_4 _in the CNS regulates airway function and inflammation in lung tissue in guinea pigs.

## Methods

### Sensitizing procedures

All animal handling was strictly in accordance with the National Institutes of Health Guide for the Care and Use of Laboratory Animals and the China Community Guidelines for the use of experimental animals. Conscious Hartley guinea pigs of either sex, weighing 400 ± 35 g, were purchased from Laboratory Animal Center of Medical College of Zhejiang University. All animals were housed in Plexiglas cages and kept on a 12/12 h light-dark cycle in temperature and humidity controlled rooms. Food was withheld 8 h before the experiments, with free access to water. Unless otherwise indicated in the text, standard laboratory food and water were provided ad libitum.

To sensitize the guinea pigs, 10 mg ovalbumin (Grade V, Sigma Chemical Co., St. Louis, MO), adsorbed in 100 mg alum aluminium hydroxide adjuvant, was intraperitonealy injected (i.p) in 1.0 ml saline and intramuscularly injected (i.m) in 0.5 ml saline into each hind leg on day 0. Negative control guinea pigs (NS-vehicle) were injected with saline following the same protocol. These animals were aerosol challenged with ovalbumin or saline on day 21 after sensitization.

### Intracerebroventricular injection

After 10% chloral hydrate (3 ml/kg i.p) anesthesia, the animal's head was fixed in a stereotaxic apparatus (SR-6N, Narishige, Japan). The procedure of i.c.v. injection was as described with minor improvement [23]. A midline incision was made from a point just posterior to the eyes to about 3 cm caudal, and the overlying connective tissue was removed to expose the skull. A hole (diameter, about 2 mm) was opened perpendicularly to the skull, -2.5 or -3.0 mm anterior and 2.5 or 3.0 mm lateral to the bregma by using a dental drill (Minimo, Japan). A stainless steel guide cannula (internal diameter, 0.5 mm; length, 1.0 cm.) was then slowly and vertically lowered to a depth of 2.5 or 3.0 mm from the dura into lateral ventricles. The guide cannula was then held in place by dental cement (oral cavity drugs and materials of Wuhan University, China) with a small anchor screw. The scalp was sutured and the animals were left to recover for 1 week before study. All injections through the i.c.v. cannula were made with a microlitre syringe (Hamilton, Reno, NV, U.S.A.) and administered in artificial CSF in a volume of 10 μl.

### Measurement of pulmonary function

Lung function was assessed as described previously [24]. Briefly, airway reactivity was determined by monitoring enhanced pause (Penh) units obtained from a single-chambered plethysmograph that measures respiratory function in unrestrained animals. The signals from the pressure transducers were continuously processed (MedLab, Nanjing Biotech Instruments, China). Ovalbumin was aerosolized into a plethysmograph from which Penh units are derived (pause 3 peak expiratory pressure/peak inspiratory pressure). Increases in Penh units, corresponding to airway reactivity to antigen in guinea pigs, was calculated as described [25]. As for antigen challenge, ovalbumin 10 mg/mL dissolved in saline was aerosolized by a jet nebulizer (BARI Co. Ltd, Germany) for 30 s 30 min after LTB_4_, vehicle or U75302 (i.c.v.) injection. To avoid anaphylactic shock, pyrilamine, an anti-histamine agent, was administered (10 mg/kg, i.p.) 30 min before the antigen challenge. Respiratory waveform was monitored for 15 min and maximal changes from baseline for each parameter were recorded by the MedLab after antigen challenge.

### Preparation of bronchoalveolar lavage fluids

Twenty-four hours after OVA challenge, guinea pigs were anesthetized with urethane (2 g/kg, i.p.), the left lung was deligated for examination of lung histopathology and LTB_4 _contents, and bronchoalveolar lavage fluids (BALF) were obtained via tracheal tube and washing of the right lung with 1.5 ml of sterilized normal saline containing 1% bovine serum albumin (BSA) and 5000 IU/l heparin for three times. Cells in the BALF were counted, the cell suspension was stained by Wright-Giemsa, and two hundred cells were classified according to cell morphology using a light microscope. The results are expressed as the numbers of each type of cell population in one ml of BALF.

### Lung histopathology

Lungs were infused via trachea with 1 ml of 10% neutral formalin. Sections of 5 μm thickness were prepared and stained with hematoxylin-eosin (H&E). To determine the severity of inflammatory cell infiltration, peribronchial eosinophil cell number was blindly counted and the severity was evaluated using a 5-point scoring system described previously [26]. Briefly, the scoring system was 5-marked, 4-moderate, 3-medium, 2-mild, 1-minimal and 0-no eosinophil cells.

### Lung and brain homogenates preparation

The procedure of lung and brain homogenates preparation was used as described in details in our previous study [21]. Briefly, after BALF, the lung artery was perfused with PBS to remove blood cells. Then the left lung and hemisphere were scissored into 1 mm × 1 mm × 1 mm cubes and homogenized in ice-cold Hanks' buffer (pH 7.5). Samples were diluted with methanol (1:1, v/v) to precipitate proteins, and centrifuged at 3500 × *g *for 10 min at 4°C. The supernatant was diluted with ultra-pure water (Water Pro Ps, LABCONCO) to obtain a final methanol concentration of 25%, and extracted on a Sep-Pak C18 column (Waters) prewashed with 20 μL of ethanol and 20 μL of water. After 200 ng PGB_2 _was added as internal standard, samples were washed through the column with 0.1% edetic acid, ultra pure water, 15% ethanol, petroleum ether and methanol in sequence. The methanolic fraction was dried under argon and stored at -80°C, and the residual mixture was dissolved in methanol before RP-HPLC assay. To minimize absorption of LTB_4_, only tubes, vials and pipette tips made of polypropylene were used. All steps of the procedure were performed under 4°C.

### Measurement of LTB_4 _content in tissue homogenization using RP-HPLC system

RP-HPLC was performed using a HP1100 separation module consisting of multiple solvent delivery systems, and equipped with UV detector, analytical pump, on-line degasser, and column thermostat. Samples were separated by a Waters symmetry C18 reversed-phase column which was protected by a Waters sentry C18 guard column. Absorbance of the column effluent was monitored using a dual wave-length absorbance detector adjusted to 270 nm for LTB_4_. Peak areas were calculated with a chromatography manager program. The mobile phase for LTB_4 _was methanol/water/acetic acid (70:30:0.01, v:v:v) adjusted to pH 5.6 with NH_4_OH. A flow rate of 1 mL/min at 35°C for LTB_4 _was used. Based on the peak areas, the LTB_4 _concentration of biological samples tested was estimated using the internal standard PGB_2_. Results are expressed as ng of LTB_4 _per g wet weight of lung or brain.

### Plasma ACTH and CORT assay

Blood samples were collected from orbital vein at two time-points: 30 min after LTB_4 _i.c.v. and 3 h after final antigen challenge. Blood samples were collected in heparin-coated tubes and centrifuged at 2000 × g at 4°C for 15 min to separate plasma. All samples were stored at -80°C until analysis. The levels of ACTH and CORT in plasma were measured using a commercial ELISA kit for guinea pig (CHZBIO, China) by following the manufacturer's instructions. The plasma was diluted 5-fold (for ACTH) or 10-fold (for CORT) with assay buffers. The detection range of guinea pig CORT was 12.2 - 600 ng/ml. The detection range of guinea pig ACTH was 12.8-200 pg/ml.

### Statistical analysis

Numerical data are presented as mean ± S.E.M. Statistical calculations were performed using SigmaStat software (SigmaStat 2.0). ANOVA and Student-Newman-Keuls multiple comparisons test were used to calculate significance of differences of respiratory function, inflammatory cells in BALF, and levels of CORT and ACTH in plasma. A non-parametric test, the Mann-Whitney U-test, was used to compare differences in eosinophil infiltration in airways. Significance was assessed at the *P *< 0.05 level.

## Results

### LTB_4 _i.c.v. attenuates antigen-induced increases in airway resistance (Penh values) in sensitized guinea pigs

**Figure 1:**
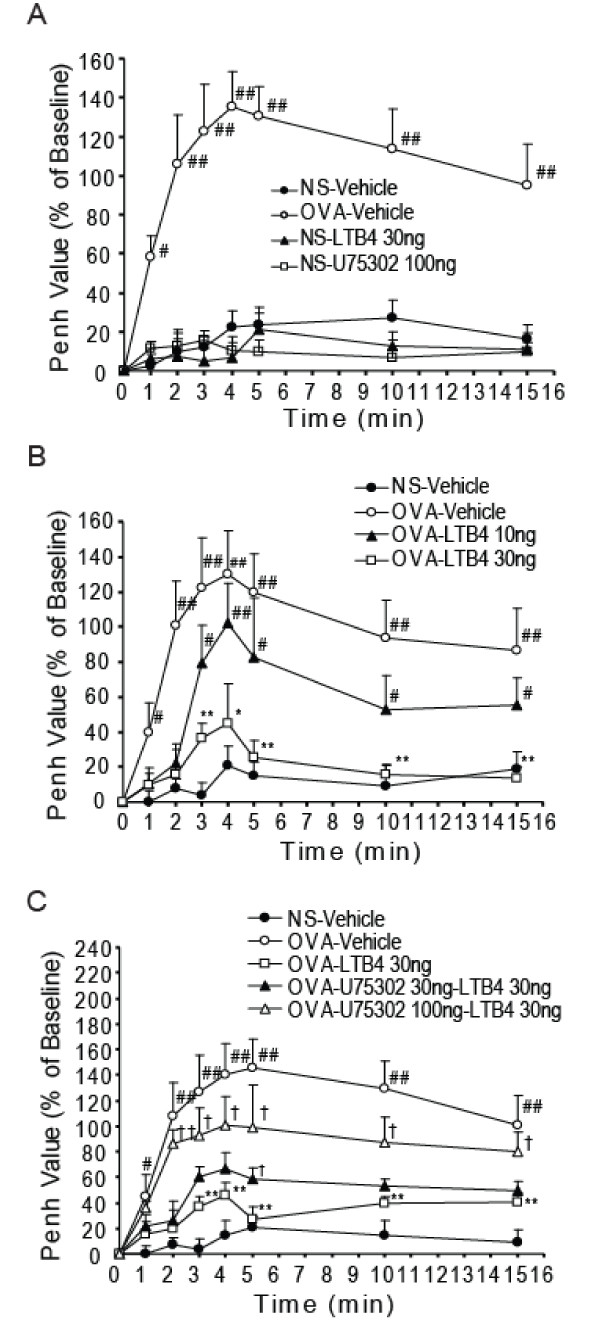
**LTB_4 _attenuates the antigen-induced asthmatic response in guinea pigs, and U75302 blocks the inhibitory effect of LTB_4_**. Animals were challenged for 30 s with aerosolized 1% ovalbumin and Penh values were measured before and 1, 2, 3, 4, 5, 10 and 15 min after the antigen challenge on day 21 after ovalbumin sensitization. Vehicle, LTB_4 _or U75302 was administered via i.c.v. before antigen challenge. Penh values are expressed as the percent change from baseline. A: LTB_4 _or U75302 i.c.v. did not increase the Penh value in saline-sensitized control guinea pigs. B: The antigen challenge-induced increase in Penh value was dose-dependently suppressed by LTB_4 _i.c.v. in ovalbumin-sensitized guinea pigs. C: U75302 100 ng i.c.v. pretreatment completely blocked the inhibitory effects of LTB_4 _on antigen-induced increases in airway resistance. Data are expressed as mean ± S.E.M. (n = 8 per group). ^#^P < 0.05, ^##^P   0.01 vs. NS-vehicle group; *P < 0.05, **P < 0.01 vs. OVA-vehicle group; †P < 0.05, ††P < 0.01 vs. OVA-LTB_4 _30 ng group. OVA, ovalbumin; NS, saline.

Compared with baseline values before aerosol saline, Penh values did not show clear increases after vehicle i.c.v. in saline-injected guinea pigs (NS-vehicle group). Similarly, in saline-injected guinea pigs, LTB_4 _or U75302 i.c.v. treatment (NS-LTB_4 _group and NS-U75302 group, respectively) did not change the Penh values. However, vehicle i.c.v. induced increases of 122% ~180% in Penh values, with maximal response after 4~5 min in ovalbumin-sensitized guinea pigs (OVA-vehicle group) (Table 1, Fig. 1A). LTB_4 _i.c.v. dose-dependently suppressed antigen challenge-induced increases in Penh values in the OVA-vehicle group (Fig. 1B). Furthermore, U75302, an LTB_4 _BLT1 receptor-selective blocker, 100 ng i.c.v. injection, almost completely blocked the inhibitory effects of LTB_4 _i.c.v. on antigen-induced increases in airway resistance in ovalbumin-sensitized guinea pigs (Fig. 1C).

**Table 1:** Time course of antigen challenge-induced changes in Penh value, and inhibitory effect of LTB_4 _via i.c.v. injection

Time (min)	% Penh value change from baseline		
	
	NS-vehicle	OVA-vehicle	OVA-LTB_4 _30 ng
0	0.0 ± 0.0	0.0 ± 0.0	0.0 ± 0.0

5	11.7 ± 9.5	179.5 ± 76.4^##^	40.2 ± 19.2 **

30	14.2 ± 8.3	61.0 ± 25.7^#^	18.8 ± 25.8 *

60	16.2 ± 17.0	28.6 ± 22.7	28.3 ± 31.6

120	8.8 ± 12.7	25.1 ± 34.6	18.2 ± 38.7

240	5.0 ± 15.2	22.2 ± 18.7	11.9 ± 22.4

1440	5.6 ± 29.5	12.6 ± 13.1	5.4 ± 30.0

Data are expressed as mean ± S.E.M. for six animals per group.

^#^*P *< 0.05, ^##^*P *< 0.01 significantly different from the NS-vehicle group;

*P < 0.05, **P < 0.01 significantly different from the OVA-vehicle group.

OVA, ovalbumin; NS, saline.

### LTB_4 _i.c.v. inhibits airway inflammatory cell appearance in BALF

**Figure 2:**
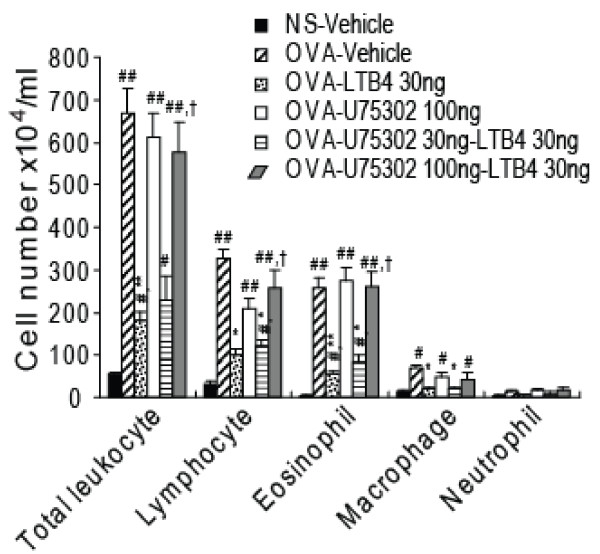
**Effects of LTB_4_, 30 ng via i.c.v. injection, on inflammatory cells in bronchoalveolar lavage fluid (BALF)**. Guinea pigs were treated as described in Methods, and BALF was harvested 24 h after OVA challenge. Total inflammatory cells in BALF were counted, and cell classification was performed on a minimum of 200 cells to classify lymphocytes, eosinophils, macrophages and neutrophils. Data are expressed as mean ± S.E.M. (n = 8 per group). ^#^*P *< 0.05, ^##^*P *< 0.01 vs. the NS-vehicle group;*P < 0.05, **P < 0.01 vs. the OVA-vehicle group; ^†^*P *< 0.05, ^††^*P *< 0.01 vs. the OVA-LTB_4 _30 ng group. OVA, ovalbumin; NS, saline.

Ovalbumin challenge (OVA-vehicle group) increased the total number of inflammatory cells in BALF to 12-fold that of saline-treated control guinea pigs (NS-vehicle group). LTB_4 _30 ng i.c.v. (OVA-LTB_4 _30 ng group) significantly decreased the total number of inflammatory cells in BALF induced by ovalbumin challenge. Classification of these inflammatory cells indicated that eosinophils, lymphocytes, macrophages and neutrophils in the BALF of ovalbumin-challenged guinea pigs increased 50-, 10-, 5- and 3.6-fold, respectively, compared with those in NS-vehicle guinea pigs (Fig. 2). LTB_4 _30 ng (i.c.v.) significantly decreased eosinophil, lymphocyte and macrophage numbers in BALF. U75302, 100 ng i.c.v., alone did not significantly affect the infiltration of inflammatory cells into airways, but did almost fully block the inhibitory effects of LTB_4_(30 ng i.c.v.) on inflammatory cell numbers in BALF. A lower dose of U75302 (30 ng, i.c.v.) did not block the effects of LTB_4 _(30 ng i.c.v.).

### LTB_4 _i.c.v. inhibits OVA-induced eosinophil infiltration in lung tissues

**Figure 3:**
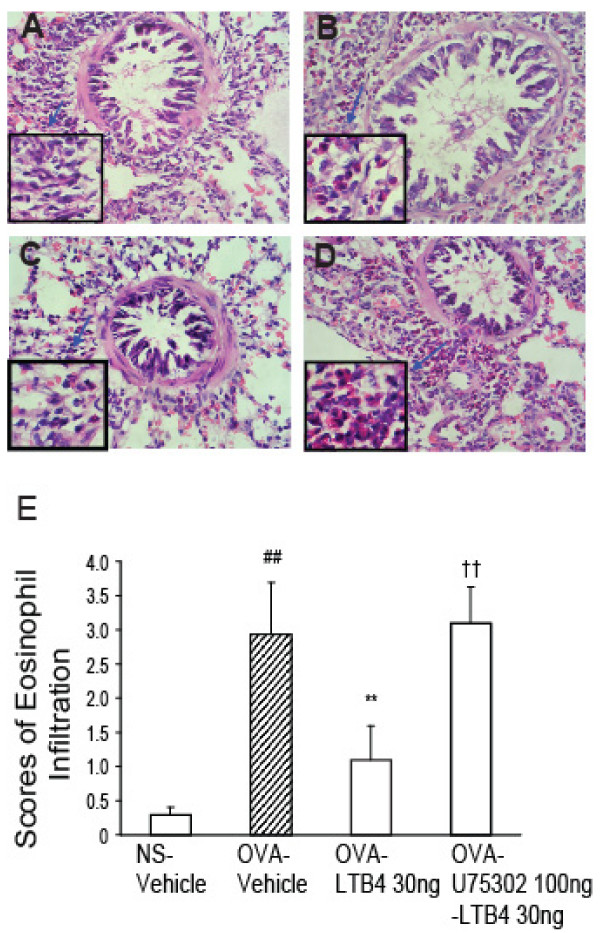
**LTB_4 _via i.c.v. suppresses antigen-induced eosinophil infiltration in lung tissue**. A-D are representative histopathological pictures of lung. Compared with NS-vehicle (A), there is a marked infiltration of eosinophil cells in the peribronchiolar space and perivascular space in the OVA-vehicle group (B). LTB_4_, 30 ng via i.c.v. injection, significantly inhibited OVA-induced eosinophil cell infiltration (C). U75302 (100 ng pretreatment via i.c.v.) blocked the inhibitory effect of LTB_4_on eosinophil cell infiltration (D). Eosinophil cell infiltration was scored based on the severity of inflammation (E). A non-parametric test, the Mann--Whitney *U*-test, was used to compare differences in eosinophil cell infiltration in lung tissues. Data are expressed as mean ± S.E.M. (n = 8 per group). ^##^*P *< 0.01 vs. the NS-vehicle group;**P < 0.01 vs. the OVA-vehicle group; ^††^*P *< 0.01 vs. the OVA-LTB_4 _30 ng group. OVA, ovalbumin; NS, saline.

Lung tissue was harvested 24 h after OVA challenge. OVA-vehicle guinea pigs exhibited an obvious eosinophil cell infiltration into the peribronchiolar and perivascular connective tissues as compared with that in NS-vehicle guinea pigs. LTB_4_, 30 ng i.c.v., markedly inhibited OVA-induced eosinophil infiltration as compared with OVA-vehicle guinea pigs. The inhibitory effect of LTB_4 _was blocked by pretreatment with U75302 via i.c.v. at a dose of 100 ng (Fig. 3).

### LTB_4 _i.c.v. has no effect on LTB_4 _content of lung and cerebral cortical homogenates from antigen-challenged asthmatic guinea pigs

**Figure 4:**
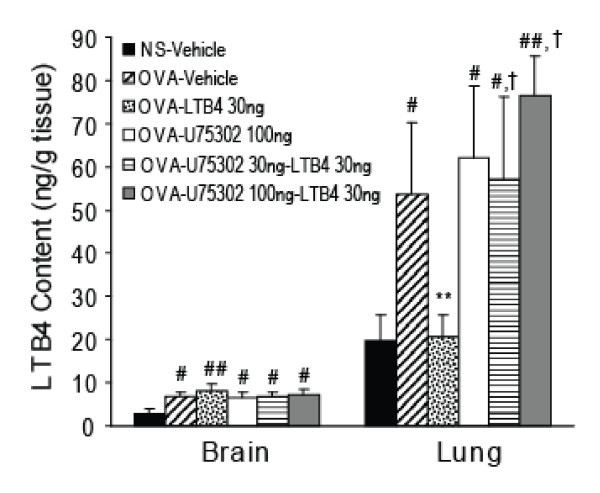
**LTB_4 _content in lung and cerebral cortex homogenates from antigen-challenged asthmatic guinea pigs**. LTB_4 _content was detected by reversed phase high-performance liquid chromatography (RP-HPLC). Data are expressed as the mean ± S.E.M. (n = 8 per group). ^#^*P *< 0.05, ^##^*P *< 0.01 vs. the NS-vehicle group;*P < 0.05, **P < 0.01 vs. the OVA-vehicle group; ^†^*P *< 0.05, ^††^*P *< 0.01 vs. the OVA-LTB_4 _30 ng group. OVA, ovalbumin; NS, saline.

The content of LTB_4 _in brain homogenates from ovalbumin-challenged guinea pigs was markedly higher than that of samples from the NS-vehicle group (*P *< 0.05). LTB_4_, 30 ng, or U75302, 100 ng, alone via i.c.v. had no effect on ovalbumin challenge-induced increases in LTB_4 _levels in brain. Neither pretreatment with 30 ng or with 100 ng U75302, 5 min before the dose of LTB_4_, 30 ng, (both via i.c.v.) had any effect on ovalbumin challenge-induced increases in LTB_4 _levels in brain. LTB_4 _levels in lung tissue homogenates from antigen-challenged guinea pigs were increased significantly compared with homogenates from saline-treated control guinea pigs (*P *< 0.05). In contrast, LTB_4_, 30 ng via i.c.v., significantly inhibited ovalbumin challenge-induced increases in LTB_4 _content of lung tissue (P < 0.01). U75302 alone at 100 ng via i.c.v. had no effect on ovalbumin challenge-induced increases of LTB_4 _content in lung tissue. However, U75302 pretreatment at doses of 30 or 100 ng via i.c.v. completely blocked the inhibitory effects of LTB_4 _(30 ng via i.c.v.) on LTB_4 _content of lung tissue (Fig. 4).

### Plasma CORT and ACTH concentrations

**Figure 5:**
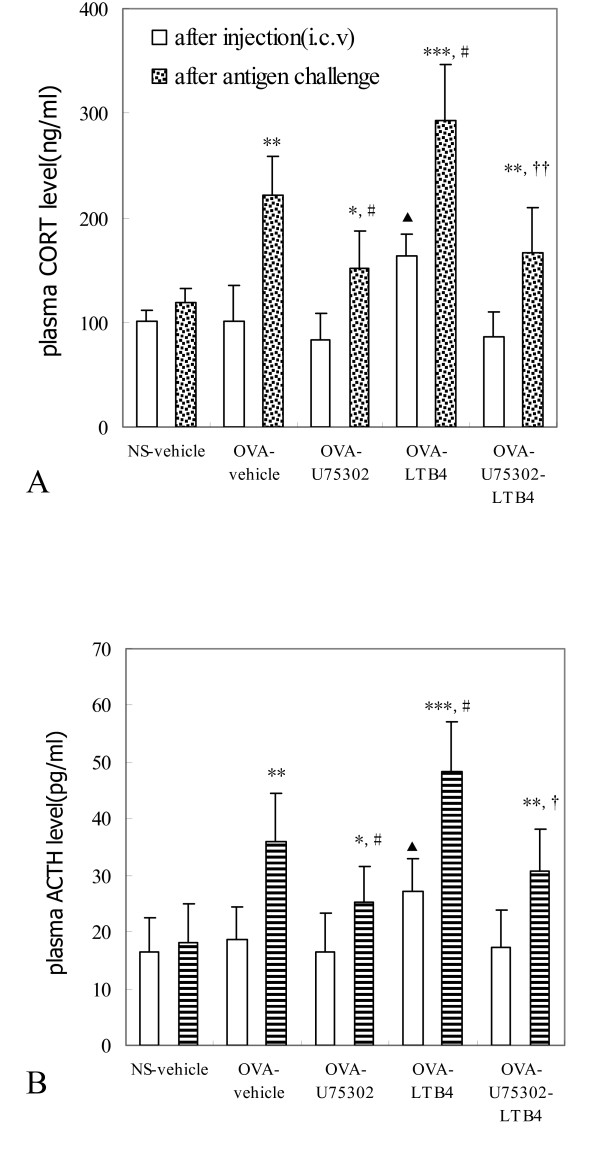
**LTB_4 _via i.c.v. injection increases plasma CORT and ACTH levels in guinea pigs**. Plasma CORT (A) and ACTH (B) levels were measured using commercial ELISA kits 30 min after either vehicle, LTB_4 _or U75302 were administered via i.c.v., both before antigen challenge and 180 min after antigen challenge. Data are expressed as mean ± S.E.M (n = 8 in each group). ANOVA and Student--Newman--Keuls multiple comparisons test were used to compare differences between groups. **P < 0.01 value is significantly different from after antigen challenge; ^#^*P *< 0.05 vs. the OVA-vehicle group after antigen challenge; *P *< 0.05 vs. the OVA-vehicle group after injection (i.c.v.); ^†^*P *< 0.05 vs. the OVA-LTB_4 _group after antigen challenge. OVA, ovalbumin; NS, saline.

To test the hypothesis that LTB_4 _exerts its inhibitory effects via the HPA axis, we measured levels of CORT and ACTH in plasma 30 min after vehicle, LTB_4_, or U75302 via i.c.v. administration, and 180 min after antigen challenge. Plasma CORT and ACTH concentrations did differ significantly after antigen challenge in all groups except for the NS-vehicle group (Fig. 5). We found that pretreatment with LTB_4 _via i.c.v. markedly increased plasma CORT and ACTH secretion rates in the LTB_4_-OVA group (*P *< 0.05) and had an additive effect after antigen challenge (*P *< 0.05), compared with OVA-vehicle. Pretreatment with U75302 (100 ng i.c.v.) produced significant decreases in plasma CORT and ACTH levels compared with OVA-vehicle after antigen challenge (*P *< 0.05). However, compared with the OVA-vehicle group, U75302 (100 ng i.c.v.) only had a partial and weak effect on the concentrations of plasma CORT (*P *> 0.05) and ACTH (*P *> 0.05) after i.c.v. injection. Furthermore, compared with LTB_4_-OVA group, pretreatment with U75302 at 100 ng suppressed LTB_4 _i.c.v.-induced increases in CORT and ACTH levels after antigen challenge (*P *< 0.05).

## Discussion

Recently, many studies have emphasized an important role for inflammatory mediators in the regulation of neuroendocrine pathways during immune challenge and in pituitary hormone secretion [27]. Particular emphasis has been placed on the cross-talk between inflammation and the HPA axis. For example, during antigen-mediated activation, CD4+ and CD8+ lymphocytes are able to produce hormones like ACTH, growth hormone (GH), thyroid stimulating hormone (TSH) and gonadotropins [28], which may regulate allergy progression. Indeed, one study has shown that an antigenic challenge delivered via either i.c.v. or i.v. routes evokes an increased HPA axis response in dogs sensitized with IgE [29]. Adrenal cortisol secretion rates increase markedly in response to antigen challenge, and evoked adrenal responses are significantly reduced by pretreatment with a histamine H1 antagonist via the i.c.v. route, but not via the i.v. route [29]. In addition, a significant attenuation of HPA axis response evoked by an antigenic challenge is observed when animals are pretreated with anti-CRF antiserum via the i.c.v. route [29]. Mast cells have long been regarded as a component of the human immune system because of their involvement in tissue-damaging and neuroimmunoendocrine modulation processes as well as in allergic and anaphylactic reactions [30]. Recent studies have indicated that the HPA axis is activated by mast cells in brain during nasal provocation in allergic rhinitis [31], and that HPA axis activation regulates cutaneous inflammatory disease [32]. However, both pharmacologic glucocorticoids and physiologic adrenal corticosteroids can ameliorate the severity of these dysfunctions and suppress the subsequent immune-mediated inflammation [22,33]. All of these studies indicate that inflammatory mediators in the CNS regulate peripheral inflammatory responses through the activation of the NEI network. Thus, the secretion of cortisol after HPA activation could conceivably evoke a life-saving host defense response against severe systemic anaphylaxis or respiratory disorders when a type I allergic reaction is triggered by antigen challenge.

LTB_4 _is a potent lipid inflammatory mediator derived from membrane phospholipids by the sequential action of cytosolic phospholipase A2, 5-LO and LTA4-H, and classically described as a chemoattractant for leukocytes [34,35]. LTB4 serves as a potent inflammatory mediator through ligation with the high affinity LTB_4 _receptor-1 (BLT1) on target cells. Many studies have shown that BLT1 is required for allergen-induced airway hyperresponsiveness and plays a role in the development of imbalance between T helper (Th)1 and Th2 cytokines during progression of asthma [36]. For example, BLT1-deleted (BLT1^-/-^) mice develop significantly lowered airway responsiveness to inhaled methacholine, lowered goblet cell hyperplasia in airways, and decreased interleukin (IL)-13 production both in lung tissue and in bronchoalveolar lavage fluid when compared with wild-type littermates [36]. Studies of allergen-induced airway hyperresponsiveness and inflammation in BLT1^-/- ^mice have shown crucial new roles for LTB_4 _and BLT1 in Th2 cytokine IL-13 production from lung Th cells, and recruitment of antigen-specific effector CD8+ T cells and CD4+ T cells [37,38], suggesting novel mechanisms for their actions in producing an imbalance in the ratio of Th1/Th2 cytokines, and a possible immune-regulation effect in asthma.

Interestingly, particularly high levels of neuronal 5-LO expression and LTB_4 _content have been identified in CNS upon challenge with a variety of stimuli [10,11,39]. The gene encoding 5-LO appears to be subject to hormonal regulation [40,41], and its neuronal expression is remarkably upregulated during aging [42], while the glucocorticoid dexamethasone inhibits 5-LO and LTA4-H mRNA expression in cerebral cortex of asthmatic rats [22]. Our previous study showed that, in addition to changes in Th1/Th2 cytokine ratios, there are also corresponding changes in LTB_4 _levels, expression of 5-LO, and LTA4-H mRNA in cerebral cortex and lung tissue in antigen-challenged asthmatic rats [21,22,25]. In this study, we found that antigen challenge induced an increase in LTB_4 _content in cerebral cortex and lung tissue in sensitized guinea pigs, which is consistent with what we previously observed in asthmatic rats [21]. In addition, we further explored the effect of increased LTB_4 _in brain on the regulation of airway inflammation and pulmonary function in asthmatic guinea pigs in this study. We found that LTB_4 _at 30 ng via i.c.v. attenuated antigen-induced airway contraction and inflammatory cell infiltration in lung tissue. U75302, a BLT1 receptor antagonist, at 100 ng via i.c.v. completely blocked the inhibitory effect of LTB_4 _on antigen-induced lung inflammation and the consequent decrease in pulmonary function. Additionally, we explored the possible mechanism for the inhibitory effect of i.c.v. LTB_4 _on inflammation and decreased pulmonary function induced by antigen in this study. We measured plasma levels of ACTH and CORT, and observed that ACTH and CORT levels in plasma increased after antigen challenge, which supports the idea that acute stress stimulation of the HPA axis is involved. Our postulation is that antigen attack provokes an acute airway response in established disease states, which may act as an acute stressor to activate the NEI system and regulate the HPA axis response.

We did not find significant differences in airway inflammation and lung mechanical function in sensitized guinea pigs treated with U75302 alone via i.c.v., which may suggest that endogenous intracerebral LTB_4 _activity does not normally play a large role in modulating airway inflammation in this model. Notably, we observed a mild decrease (around 15%-30%) in ACTH and CORT levels in plasma after U75302 block of the endogenous LTB_4 _receptor. We postulate that the increased endogenous LTB_4 _induced by antigen challenge may mildly activate the HPA axis, but that this activation of the HPA axis may be not enough to antagonize peripheral inflammation in this asthmatic model. Another possible explanation is that the functional effect of increased endogenous LTB_4 _induced by antigen challenge may be balanced by other mediators or cytokines in brain. For example, levels of TNF-α, IL-1 and IL-6 during asthmatic attack in brain are also changed after antigen challenge. Further studies are needed to clarify how the HPA axis responds to changes in asthma-related cytokines and other inflammatory mediators, and how the HPA axis communicates with neural and endocrine networks as well as their signal pathways in regulating peripheral allergic responses.

## Conclusion

This study finds that LTB_4_, administered via i.c.v., attenuates pulmonary inflammation and decreases lung function changes induced by antigen challenge in sensitized guinea pigs via a mechanism involving the BLT1 receptor. This study expands our concept of the regulatory role of intracranial inflammatory mediators in inflammatory diseases including asthma, and suggests a link between intracranial LTB_4 _and neuroendocrine networks. This study also suggests that increases in LTB_4 _levels are involved in the pathophysiology of allergy, regardless of the target organ affected, and appear to be part of a negative feedback regulation system associated with corticosterone production resulting from activation of the HPA axis. In line with this concept, these inflammatory factors probably have some favorable effects on the HPA axis of asthmatics, and may help to explain the phenomenon of self-relief after an asthmatic attack.

## Competing interests

The authors declare that they have no competing interests.

## Authors' contributions

YLZ conducted the study and participated in study design, analyzed data and prepared the figures. YMD and QMX designed the study, analyzed data and wrote the manuscript. YLZ, SJZ ran the HPLC analysis and assay of plasma adrenocorticotropic hormone and corticosterone, and YLZ, YMD, XWD, SJZ, and JXJ performed the pulmonary function and lung pathological evaluation. All authors have read and approved the final manuscript.
